# Expression profiles of meiotic genes in male vs. female gonads and gametes: Insights into fertility issues

**DOI:** 10.3389/fgene.2023.1125097

**Published:** 2023-03-14

**Authors:** Marília Körbes Rockenbach, Lucas Rosa Fraga, Thayne Woycinck Kowalski, Maria Teresa Vieira Sanseverino

**Affiliations:** ^1^ Postgraduate Program in Genetics and Molecular Biology, Universidade Federal do Rio Grande do Sul (UFRGS), Porto Alegre, Brazil; ^2^ Department of Morphological Sciences, Institute of Health Sciences, Universidade Federal do Rio Grande do Sul (UFRGS), Porto Alegre, Brazil; ^3^ Postgraduate Program in Medicine: Medical Sciences, Universidade Federal do Rio Grande do Sul (UFRGS), Porto Alegre, Brazil; ^4^ Laboratory of Genomic Medicine, Center of Experimental Research, Hospital de Clínicas de Porto Alegre, Porto Alegre, Brazil; ^5^ Bioinformatics Core, Hospital de Clínicas de Porto Alegre, Porto Alegre, Brazil; ^6^ Medical Genetics Service, Hospital de Clínicas de Porto Alegre, Porto Alegre, Brazil; ^7^ Centro Universitário CESUCA, Cachoeirinha, Brazil; ^8^ School of Medicine, Pontifícia Universidade Catolica do Rio Grande do Sul, Porto Alegre, Brazil

**Keywords:** oogenesis, spermatogenesis, germinal vesicle, mature oocyte, infertility

## Abstract

Gametes are specialized cells that, at fertilization, give rise to a totipotent zygote capable of generating an entire organism. Female and male germ cells undergo meiosis to produce mature gametes; however, sex-specific events of oogenesis and spermatogenesis contribute to specific roles of gametes in reproductive issues. We investigate the differential gene expression (DGE) of meiosis-related genes in human female and male gonads and gametes in normal and pathological conditions. The transcriptome data for the DGE analysis was obtained through the Gene Expression Omnibus repository, comprising human ovary and testicle samples of the prenatal period and adulthood, additionally to male (non-obstructive azoospermia (NOA) and teratozoospermia), and female (polycystic ovary syndrome (PCOS) and advanced maternal age) reproductive conditions. Gene ontology terms related to meiosis were associated with 678 genes, of which 17 genes in common were differentially expressed between the testicle and ovary during the prenatal period and adulthood. Except for *SERPINA5* and *SOX9*, the 17 meiosis-related genes were downregulated in the testicle during the prenatal period and upregulated in adulthood compared to the ovary. No differences were observed in the oocytes of PCOS patients; however, meiosis-related genes were differentially expressed according to the patient’s age and maturity of the oocyte. In NOA and teratozoospermia, 145 meiosis-related genes were differentially expressed in comparison to the control, including *OOEP*; despite no recognized role in male reproduction, *OOEP* was co-expressed with genes related to male fertility. Taking together, these results shed light on potential genes that might be relevant to comprehend human fertility disorders.

## 1 Introduction

Germ cells undergo a series of mitotic and meiotic divisions, followed by a differentiation program to produce highly specialized haploid gametes that, at fertilization, fuse and give rise to a totipotent zygote, capable of generating all somatic lineages/tissues and the next-generation of gametes (Larose et al., 2019; Farini and De Felici, 2022). The proper completion of meiosis is essential for fertility and for ensuring the normal development of the offspring (Farini and De Felici, 2022). Therefore, revealing the molecular mechanisms and pathways underlying this unique process is pivotal for understanding human reproduction and fertility, as well as helping to identify therapeutic and diagnostic targets (Larose et al., 2019; Farini and De Felici, 2022).

Infertility affects 8%–12% of reproductive-age couples worldwide (Ombelet et al., 2008), reaching 30% in some regions of the world (Mascarenhas et al., 2012). In 33% of infertility cases, the inability to have children is due to female factors alone, in 20% to male factors, in 39% to both male and female factors, and in 8% the causes remain unknown, termed as unexplained infertility (Thonneau et al., 1991). Understanding meiosis may reveal possible mechanisms underlying the cases of unexplained infertility, since proper gametogenesis is important to the formation of mature gametes and to guarantee the first events of embryo development (Larose et al., 2019).

Both female and male germ cells undergo meiosis; however, the timing of meiosis and the quantity of gametes that are produced differs in males and females (Farini and De Felici, 2022). In mammals, meiosis begins during fetal life in females and continues until menopause, whilst in males it begins at puberty and is maintained throughout life (Farini and De Felici, 2022). Understanding the molecular mechanisms behind the differences in meiosis onset and progression in males and females may contribute to the elucidation of genes involved in conditions affecting human reproduction.

The current study aimed to investigate if the gene expression profile of meiosis-related genes in human male and female gonads and gametes could be associated with human reproductive disorders. To achieve that, the expression of 678 meiosis-related genes was evaluated in normal and pathological conditions affecting human reproduction, through publicly available datasets. The interesting pattern of expression demonstrated in this study sheds light on the importance of gene expression regulation programs in human reproductive disorders and elucidates potential biomarkers in the reproductive medicine field.

## 2 Methods

### 2.1 Search for ontologies and genes related to meiosis

**FIGURE 1:**
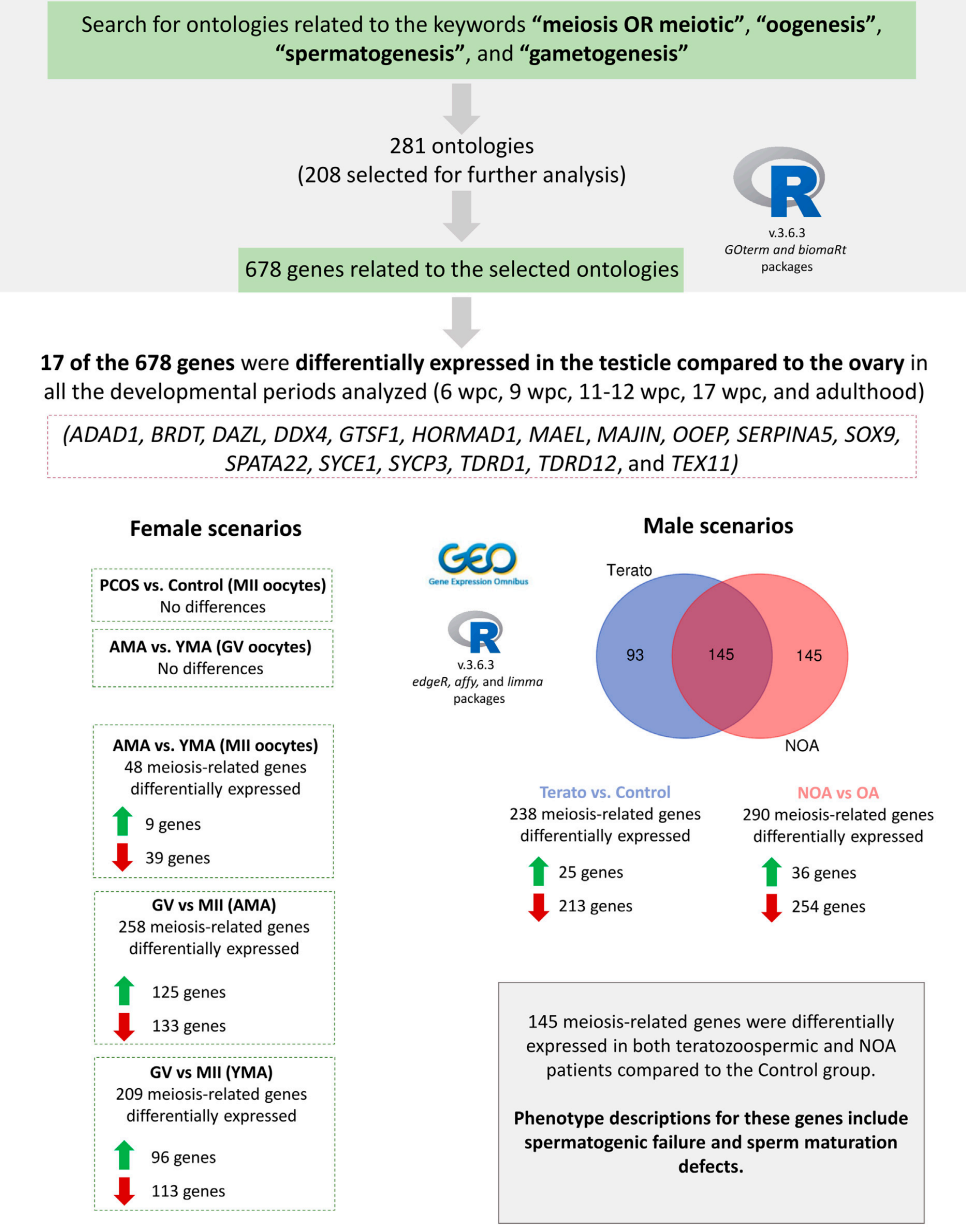
Study design and main results found. A search for gene ontology (GO) terms related to the meiosis process was conducted in the R software, resulting in 281 GO terms. Of these, 208 were selected, being further associated with 678 meiosis-related genes. Six datasets were selected in the Gene Expression Omnibus (GEO) repository for differential gene expression analysis. Considering the 678 meiosis-related genes, 17 were differentially expressed in the testicle compared to the ovary in both the prenatal period and adulthood. No statistically significant differences were observed in MII oocytes of PCOS patients in comparison to the Control group, nor in GV oocytes of AMA in comparison to YMA patients. However, 48 meiosis-related genes were differentially expressed in MII oocytes of AMA in comparison to YMA patients. Besides, when comparing GV vs. MII oocytes, 258 and 209 meiosis-related genes were differentially expressed in AMA and YMA patients, respectively. In male reproductive disorders’ scenario, 238 and 290 meiosis-related genes were differentially expressed in teratozoospermic and NOA patients in comparison to the Control groups, respectively, of which 145 genes were differentially expressed in both conditions. Green up arrows mean upregulated genes, whilst red down arrows downregulated genes. wpc (weeks post-conception); PCOS (Polycystic Ovary Syndrome); AMA (advanced maternal age); YMA (young maternal age); GV (germinal vesicle); MII (mature oocytes at metaphase II stage); NOA (non-obstructive azoospermia); OA (obstructive azoospermia).

The entire study was performed through bioinformatic analysis of publicly available data, divided in two stages: (1) Search for genes related to meiosis, and (2) Gene expression analysis of meiosis-related genes in human gonads and gametes (Figure 1). To obtain the genes related to meiosis for secondary analysis of gene expression, an ontology-based search was conducted in the R software (v.3.6.3). Gene ontology (GO) terms related to meiosis were selected using the GOterm function from the *AnnotationDBI* R package (v.1.48.0) and the following keywords: “meiosis OR meiotic”, “oogenesis”, “spermatogenesis”, and “gametogenesis”. A list containing all the GO terms retrieved (Supplementary Table S1) was evaluated by two authors (MKR and TWK), independently. Only GO terms associated with human gametogenesis were included. GO terms selected by both authors were included; when one GO term was selected by only one author, the selection was discussed and a consensus was reached. The genes associated with the selected ontologies were obtained through the *biomaRt* package (v.2.42.0), considering the human reference genome (hg38).

Further information regarding the selected genes functions and clinical associations were obtained through the GeneCards and Hugo Gene Nomenclature Committee (HGNC) databases (Stelzer et al., 2016; Tweedie et al., 2021), as well as through the *biomaRt* package (v.2.42.0), filtering by human phenotype descriptions available in the Ensembl database (Cunningham et al., 2022).

### 2.2 Obtainment and comparisons of transcriptome data

To analyze the expression of meiosis-related genes in human reproductive disorders, we performed differential gene expression (DGE) analysis in transcriptomic data available in the Gene Expression Omnibus (GEO) repository (NCBI, USA) (Edgar et al., 2002; Barrett et al., 2013). The keywords “oocyte”, “oogonia”, “mature oocyte”, “immature oocyte”, “germinal vesicle”, “ovary”, “oogenesis”, “gametogenesis”, “sperm” “spermatozoa”, “spermatid”, “spermatocyte”, “spermatogonia”, “spermatogenesis”, and “testes OR testicle” were used for the datasets search, filtering by Entry type (Series), organism (*Homo sapiens*), and study type (Expression Profile by Array or Expression Profile by Throughput Sequencing). Only studies performed in consolidated platforms, containing the raw data, experimental design and sample groups available were included. For microarrays, customized libraries without probe correspondence were also excluded. Following these criteria, six datasets were selected for the DGE analysis, comprising human samples of male and female gonads, mature and immature oocytes, and sperm, under normal and reproductive pathological conditions. The datasets are publicly available in the GEO repository, registered as GSE7307 (Hever et al., 2007), GSE116278 (Lecluze et al., 2020), GSE5850 (Wood et al., 2007), GSE164371 (Ntostis et al., 2021), GSE6872 (Platts et al., 2007), and GSE145467 (Hodžić et al., 2021). Additional information about the selected datasets for DGE analysis is available in (Supplementary Table S2).

The meiosis-related genes obtained from the gene ontology-based search (678 genes) had their expression compared between the testicle and ovary samples in five different life stages (6, 9, 11–12, 17 weeks post-conception (wpc), and adult), and in conditions affecting human reproduction. The comparison of the expression in the testicle vs. ovary was performed separately for each of the five stages. Regarding conditions affecting female fertility, the DGE of *in vivo* matured metaphase II (MII) oocytes in Polycystic Ovary Syndrome (PCOS) *versus* Control patients was analyzed, as well as in MII and germinal vesicle (GV) oocytes of advanced maternal age (41–44 years old) and young maternal age (21–26 years old) women. Concerning male reproductive disorders, the DGE was analyzed in sperm samples of Teratozoospermic (≤3% of spermatozoa displaying the ideal form) *versus* Normospermic patients, as well as in testicle samples of patients with non-obstructive azoospermia (NOA–impaired spermatogenesis) *versus* obstructive azoospermia (OA–normal spermatogenesis).

### 2.3 Differential gene expression analysis

DGE analysis was conducted in the R environment (v.3.6.3). Firstly, for RNA-Seq data, the alignment was performed through the Galaxy Europe server (Jalili et al., 2020), using the HISAT2 (Kim et al., 2019) alignment tool against human reference genome hg38; transcript count was performed through featureCounts tool (Liao et al., 2014). The mapping with HISAT2 and transcript count with featureCounts were based on a standardized workflow from our Bioinformatics Core, which one of the authors (TWK) is part of. The parameters for RNA-Seq data alignment and transcript count were the default ones, and the alignment rate was above 80% for all the samples analyzed. The DGE was accessed in the aligned transcriptomes using the *edgeR* (v.3.28.1) (Robinson et al., 2010) package. Considering microarray data, the packages *affy* (v.1.64.0) (Gautier et al., 2004) and *limma* (v.3.42.2) (Ritchie et al., 2015) were used to normalize and evaluate the DGE. RNA-Seq data was normalized through the trimmed mean of M values (TMM) and microarray data by robust multi-array average (RMA).

### 2.4 Systems biology analysis

In the light of the DGE results, a gene-gene co-expression network for *OOEP,* a member of the Subcortical Maternal Complex (SCMC), was performed in the Cytoscape software (v.3.8) using the GeneMania application (Montojo et al., 2010). Only co-expression data was considered.

### 2.5 Statistical analysis

Statistical analysis were performed in the R environment (v.3.6.3). The results are demonstrated as values of log_2_ fold-change (logFC), and adjusted *p*-value for false discovery rate (FDR). The logFC measures the ratio between two quantities in a base 2 logarithmic scale (for instance, a doubling is equivalent to a logFC of 1, a quadrupling to a logFC of 2, and so on). Similarly, negative logFC values mean decreased amounts. The DGE was considered significant when identifying a gene with both a logFC ≥ |1| and an adjusted *p*-value ≤0.05.

## 3 Results

### 3.1 Ontologies and genes related to meiosis

The search terms were associated with 281 ontologies, being selected 208 for further analysis (Supplementary Table S1); Although GO includes three independent categories: molecular function, biological process, and cellular component, our keywords only returned GO terms related to biological processes. 678 genes were related to these ontologies (Supplementary Table S3), mostly involved in mechanisms of DNA repair, chromosome segregation, and meiotic regulation. Of the 678 meiosis-related genes, 243 had clinical associations available in the Ensembl database (Supplementary Table S4), including associations to spermatogenic failure, sperm motility disorder, oocyte meiotic arrest, and oocyte maturation defect.

### 3.2 Differential expression of meiosis-related genes under normal and pathological human reproductive conditions

**FIGURE 2:**
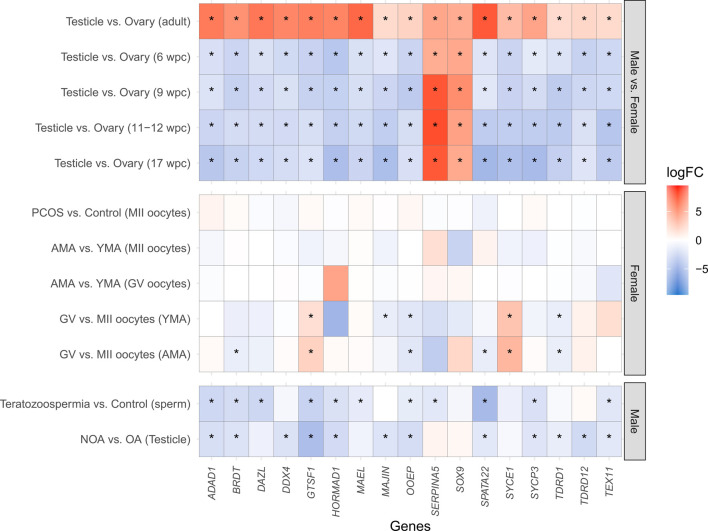
Heatmap of the differential gene expression of the 17 meiosis-related genes differentially expressed in all the developmental periods analyzed. The *X*-axis indicates the analyzed genes, whilst the *Y*-axis represents the comparisons performed. Except for *SERPINA5* and *SOX9*, which maintain the upregulation in the testicle in both prenatal and adult periods, all the other genes were downregulated in the testicle in comparison to the ovary during the prenatal periods (6 wpc, 9 wpc, 11–12 wpc, and 17 wpc), and upregulated in the adulthood. No statistically significant differences were observed in MII oocytes of PCOS patients in comparison to the Control group, neither in MII and GV oocytes of AMA in comparison to YMA patients. However, when comparing GV vs. MII oocytes, a downregulation of *OOEP* and *TDRD1*, as well as an upregulation of *GTSF1* and *SYCE1*, was observed in both AMA and YMA patients. A downregulation of *MAJIN* in GV in comparison to MII oocytes was observed only in YMA, and a downregulated of *BRDT* and *SPATA22* in AMA. A downregulation of *ADAD1, BRDT, GTSF1, HORMAD1, OOEP, SPATA22, SYCP3*, and *TEX11* in both teratozoospermic and NOA patients in comparison to the Control groups. A downregulation of *DAZL*, *MAEL*, and *SERPINA5* was demonstrated only in teratozoospermic patients, as well as a downregulation of *DDX4*, *MAJIN*, *TDRD1*, and *TDRD12* in NOA patients, in comparison to the Control groups. wpc (weeks post-conception); PCOS (Polycystic Ovary Syndrome); AMA (advanced maternal age); YMA (young maternal age); GV (germinal vesicle); MII (mature oocytes at metaphase II stage); NOA (non-obstructive azoospermia–impaired spermatogenesis); OA (obstructive azoospermia–normal spermatogenesis); logFC (Log_2_ Fold Change); * means statistically significant differences between groups (logFC ≥ |1| and adjusted *p*-value ≤0.05). Warm colors (red) represent upregulated genes, whilst cool colors (blue) downregulated genes.

The DGE of the 678 genes related to meiosis was assessed in human male and female gonads according to the life period (in adulthood and at 6, 9, 11–12, and 17 wpc). Of the 678 genes, 17 (*ADAD1, BRDT, DAZL, DDX4, GTSF1, HORMAD1, MAEL*, *MAJIN, OOEP, SERPINA5, SOX9, SPATA22, SYCE1, SYCP3, TDRD1, TDRD12*, and *TEX11*) were differentially expressed in the testicle in comparison to the ovary in all the analyzed developmental periods. In the adult gonads, more meiosis-related genes were upregulated in the testicle in comparison to the ovary, whilst in all the prenatal periods the meiosis genes were mostly upregulated in the ovary. The same pattern was observed for the 17 differentially expressed genes in all developmental periods, except for *SERPINA5* and *SOX9,* which maintain the upregulation in the testicle in both prenatal and adults periods, as expected considering their roles in male reproduction (Yang and Geiger, 2017; Selvam et al., 2019; Jiménez et al., 2021) (Figure 2). These 17 genes are mainly related to processes involved in homologous chromosome pairing, recombination, and segregation (Supplementary Table S5).

No statistically significant differences were observed in MII oocytes of PCOS patients in comparison to the Control group, nor in GV oocytes of advanced maternal age in comparison to young maternal age patients. However, 48 meiosis-related genes were differentially expressed in MII oocytes of advanced maternal age in comparison to young maternal age patients. Besides, when comparing GV vs. MII oocytes, 258 and 209 meiosis-related genes were differentially expressed in advanced maternal age and young maternal age patients, respectively.

In view of male reproductive disorders, 238 and 290 meiosis-related genes were differentially expressed in teratozoospermic and NOA patients in comparison to the control groups, respectively, of which 145 genes were differentially expressed in both conditions. Interestingly, the majority of the meiosis-related genes were downregulated in both teratozoospermic (213 genes) and NOA (254 genes) patients compared to the control samples.

Considering only the 17 differentially expressed genes between male and female gonads in all the developmental periods, a downregulation of *OOEP* and *TDRD1*, as well as an upregulation of *GTSF1* and *SYCE1*, was observed in MII oocytes of both advanced maternal age and young maternal age patients. A downregulation of *MAJIN* in GV was observed only in young maternal age, and a downregulated of *BRDT* and *SPATA22* in advanced maternal age. In male fertility disorders, it was observed downregulation of *ADAD1, BRDT, GTSF1, HORMAD1, OOEP, SPATA22, SYCP3*, and *TEX11* in both teratozoospermic and NOA patients in comparison to the Control groups. A downregulation of *DAZL*, *MAEL*, and *SERPINA5* was demonstrated only in teratozoospermic patients, as well as a downregulation of *DDX4*, *MAJIN*, *TDRD1*, and *TDRD12* in NOA patients, in comparison to the Control groups (Figure 2).

The logFC and the adjusted *p*-Values of all the statistical analysis are available in (Supplementary Table S6).

### 3.3 Co-expression network for *OOEP* gene

**FIGURE 3:**
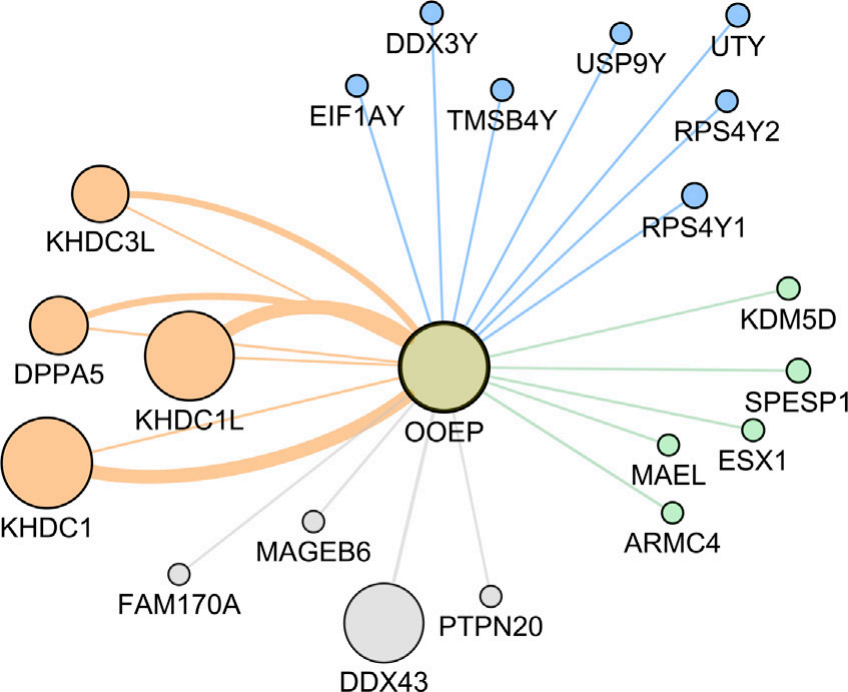
Oocyte expressed protein (OOEP) gene-gene co-expression network. The edges connecting *OOEP* with other genes represent co-expression, and larger circles mean stronger evidence of gene co-expression. Orange means genes related with human reproduction; gray: no associated with human reproduction; green: related with spermatogenesis and/or male infertility; blue: located in the Y chromosome. *DPPA5* (Developmental Pluripotency Associated 5); *KHDC1* (KH Domain Containing 1); *KHDC1L* (KH Domain Containing 1 Like); *KHDC3L* (KH Domain Containing 3 Like); *FAM170A* (Family With Sequence Similarity 170 Member A); *DDX43* (DEAD-Box Helicase 43); *MAGEB6* (MAGE Family Member B6); *PTPN20* (Protein Tyrosine Phosphatase Non-Receptor Type 20); *ARMC4* (Armadillo Repeat-Containing Protein 4); *ESX1* (ESX Homeobox 1); *KDM5D* (Lysine Demethylase 5D); *MAEL* (Maelstrom Spermatogenic Transposon Silencer); *SPESP1* (Sperm Equatorial Segment Protein 1); *EIF1AY* (Eukaryotic Translation Initiation Factor 1A Y-Linked); *DDX3Y* (DEAD-Box Helicase 3 Y-Linked); *RPS4Y1* (Ribosomal Protein S4 Y-Linked 1); *RPS4Y2* (Ribosomal Protein S4 Y-Linked 2); *TMSB4Y* (Thymosin Beta 4 Y-Linked); *USP9Y* (Ubiquitin Specific Peptidase 9 Y-Linked); *UTY* (Ubiquitously Transcribed Tetratricopeptide Repeat Containing, Y-Linked).

According to the literature review performed by the completion of this manuscript, *OOEP* has no recognized role in male reproduction. Therefore, considering the downregulation of *OOEP* observed in male reproductive disorders, a co-expression network was performed for this gene to better understand the involvement of *OOEP* in male reproduction. It demonstrated the co-expression of *OOEP* with genes related to human reproduction, spermatogenesis, male infertility, and located in the Y chromosome (Figure 3). The main functions and reproductive disorders associated with these genes are shown in (Supplementary Table S7).

## 4 Discussion

Both female and male gametogenesis begin with the mitotic proliferation of oogonia and spermatogonial stem cells, respectively, followed by meiotic divisions and modifications until the formation of mature germ cells (Larose et al., 2019). However, the timing and quantity of gametes developed are very different in the two gonads, and gene expression patterns may govern, at least in part, these differences (Handel and Schimenti, 2010). In males, the spermatogonial stem cells remain quiescent during the prenatal period, starting to proliferate and entering meiosis after puberty, resulting in continuous sperm production throughout the reproductive lifespan (Handel and Schimenti, 2010). On the other hand, female meiosis initiates in fetal ovaries and is arrested in the prophase I stage before birth, being reestablished only after puberty in periodical intervals in response to hormonal stimuli (Handel and Schimenti, 2010). To accomplish these processes, intracellular signals, such as the cAMP levels in the oocyte, are necessaire for the maintenance of meiotic arrest or for the meiotic resumption, which is characterized by the germinal vesicle (GV) breakdown, followed by completion of meiosis I, and arrest at the metaphase of meiosis II until fertilization (Pan and Li, 2019). Hence, differences in gene expression in the GV (prophase I-arrested oocytes) and MII (mature oocytes), as demonstrated in the present study, may be related to molecular mechanisms involved in the regulation of meiotic arrest and resumption; these molecular mechanisms might also be related to the acquisition of oocyte competence, pivotal to support the early embryo development (Conti and Franciosi, 2018).

Complex biological processes, such as those involved in human reproduction, require different genes working in an orchestrated way to ensure regulatory networks (de la Fuente, 2010). Recent studies of global gene expression in oocytes and sperm have demonstrated important target genes involved in conditions affecting human reproduction (Hermann et al., 2018; Li et al., 2021; Ntostis et al., 2021; Santiago et al., 2021; Sayutti et al., 2022). In the present study, prior filtering of meiosis-related genes was performed, and these genes had their differential gene expression evaluated in female and male gonads and gametes under physiological and pathological conditions.

**FIGURE 4:**
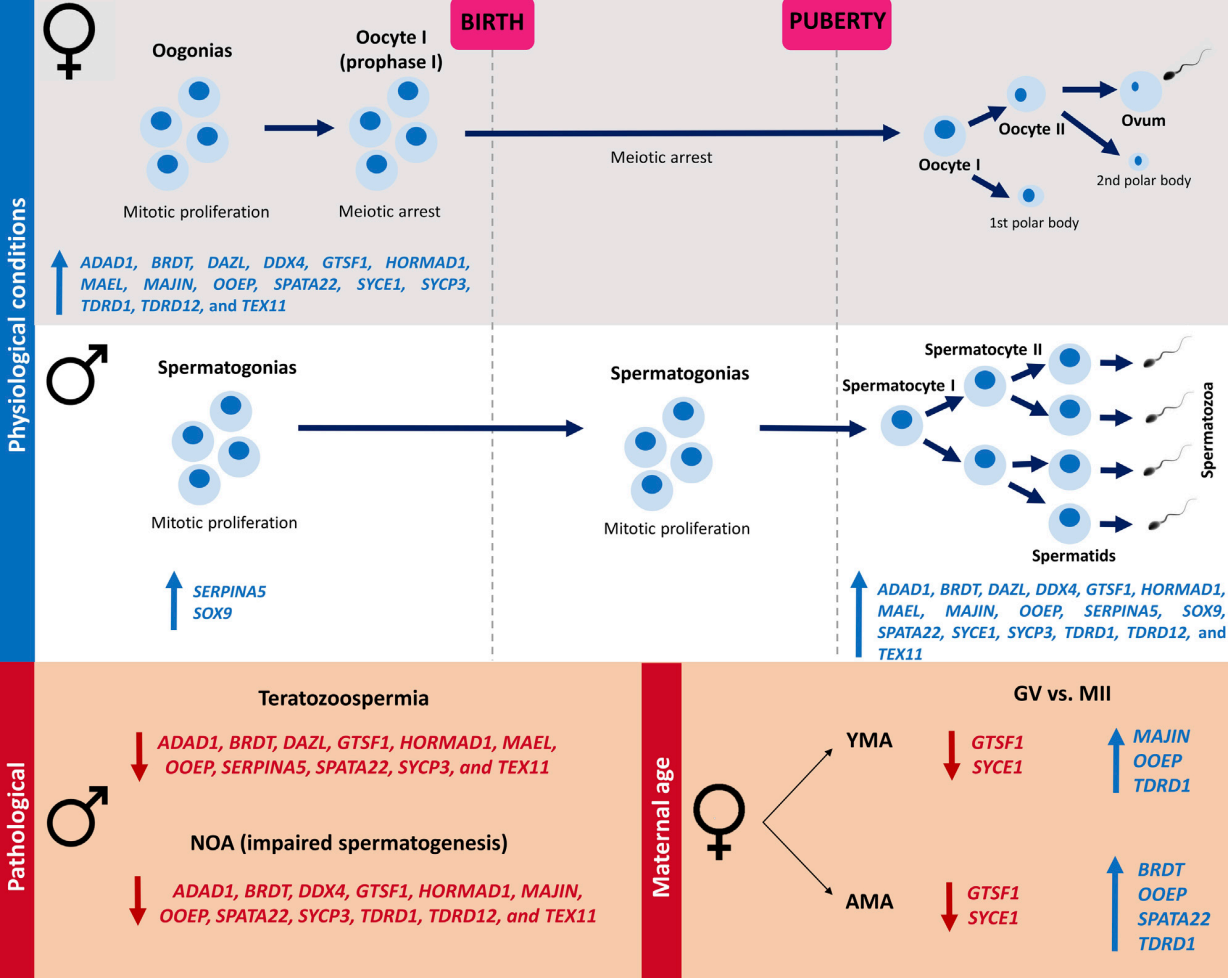
Illustration of the 17 meiosis-related genes expression in human ovary and testicle according to the life period and gametogenesis timing under physiological and pathological scenarios. Female meiosis initiates in fetal ovaries and is arrested in the prophase I stage before birth, being reestablished after puberty in periodical intervals in response to hormonal stimuli. In males, the spermatogonial stem cells remain quiescent during the prenatal period, starting to proliferate and entering meiosis after puberty, resulting in continuous sperm production throughout the reproductive lifespan. It was demonstrated that during adulthood, 17 meiosis-related genes were upregulated in the testicle in comparison to the ovary (*ADAD1, BRDT, DAZL, DDX4, GTSF1, HORMAD1, MAEL*, *MAJIN, OOEP, SERPINA5, SOX9, SPATA22, SYCE1, SYCP3, TDRD1, TDRD12*, and *TEX11)*, whilst in all the prenatal periods analyzed, downregulation of these genes was observed in the testicle in comparison to the ovary, except by *SERPINA5* and *SOX9*, that maintain the upregulation in the testicle. It was observed a downregulation of *ADAD1, BRDT, GTSF1, HORMAD1, OOEP, SPATA22, SYCP3*, and *TEX11* in both teratozoospermic and NOA patients in comparison to the Control groups. A downregulation of *DAZL*, *MAEL*, and *SERPINA5* was demonstrated only in teratozoospermic patients, as well as a downregulation of *DDX4*, *MAJIN*, *TDRD1*, and *TDRD12* in NOA patients, in comparison to the Control groups. Comparing GV vs. MII oocytes, a downregulation of *OOEP* and *TDRD1*, as well as an upregulation of *GTSF1* and *SYCE1*, was observed in MII oocytes of both AMA and YMA patients. A downregulation of *MAJIN* in GV was observed only in YMA, and a downregulated of *BRDT* and *SPATA22* in AMA. NOA (non-obstructive azoospermia); GV (germinal vesicle); MII (mature oocytes at metaphase II stage); YMA (young maternal age); AMA (advanced maternal age); blue up arrows mean upregulated genes, whilst red down arrows mean downregulated genes.

It was demonstrated that during adulthood, 17 meiosis-related genes were upregulated in the testicle in comparison to the ovary (*ADAD1, BRDT, DAZL, DDX4, GTSF1, HORMAD1, MAEL*, *MAJIN, OOEP, SERPINA5, SOX9, SPATA22, SYCE1, SYCP3, TDRD1, TDRD12*, and *TEX11*), whilst in all the prenatal periods analyzed, downregulation of these genes was observed in the testicle in comparison to the ovary, except for *SERPINA5* and *SOX9*, that maintain the upregulation in the testicle. The orchestrated expression of these genes during meiosis, according to time and place, may be crucial for meiosis entry and normal gamete formation (Figure 4), considering their involvement in important processes such as: meiotic regulation, homologous chromosomes pairing, recombination and segregation, DNA double-strand breaks (DSBs) repair, and formation of the synaptonemal complex (GeneCards, 2022). In addition, considering the 678 meiosis-related genes, it was observed that more genes were upregulated in the ovary during the prenatal period compared to the testicle, whilst in the adult gonads more genes related to meiosis were upregulated in the testicle.

During the meiosis prophase I, the synaptonemal complex mediates the homologous chromosomes pairing and synapsis, a fundamental step for the recombination, that is initiated by DNA double strand breaks, and the segregation events (Handel and Schimenti, 2010). In human fetal ovaries, meiotic entry is initiated asynchronously around 8-9 wpc, confirmed by the increased expression of meiotic proteins by this time, such as DAZL, DDX4, and SYCP3 (Farini and De Felici, 2022); this increased expression was corroborated in the present study. The conditions for meiotic beginning depend on intrinsic and extrinsic factors, being proposed that germ cells of both sexes receive at the same time stimuli for meiotic beginning, however, somatic cells in the developing gonads induce or prevent meiosis entry and progression (Farini and De Felici, 2022). Therefore, the distinct pattern of meiosis-related gene expression observed in the testicle and ovary according to the life period may reflect the differences in female and male meiosis onset, through differential gene expression of germ and somatic cells in fetal and adult gonads. The upregulation of the 17 meiosis-related genes in the fetal ovary and adult testicle, coinciding with the meiosis onset in both sexes, is in agreement with the roles of these genes in processes mainly related to prophase I, the first stage of meiosis.

*SERPINA5* and *SOX9* were upregulated in the testicle in both prenatal and adult periods. SOX9 is a transcription factor expressed in the Sertoli cells of the male gonad throughout life (Jiménez et al., 2021). At the time of sex determination, the expression of the Y-linked, sex determining-region - *SRY* - in the Sertoli cells leads to the upregulation of *SOX9*, which activates male-promoting genes necessary for the differentiation of Sertoli cells into the testicle cords (Jiménez et al., 2021). *SERPINA5* encodes a glycoprotein with an inhibitory effect in several serine proteases; in seminal plasma, inactivates several serine proteases implicated in the reproductive system, and has a control on the sperm motility and fertilization (GeneCards, 2022). Although the roles of *SERPINA5* are poorly understood (Yang and Geiger, 2017), studies have pointed to a role in male reproduction (Yang and Geiger, 2017; Selvam et al., 2019). Therefore, the constant upregulation of *SOX9* and *SERPINA5* in the testicle samples in comparison to the ovary is in agreement with their roles in processes related to male reproduction.

Chromosome segregation errors during meiosis, leading to aneuploidies, are one of the most common causes of infertility, pregnancy failure, and genetic disorders in the offspring (Mikwar et al., 2020). Although meiotic errors may occur in both male and female germ cells, the majority are of maternal origin, being the advanced maternal age (≥35 years) a risk factor for chromosome segregation errors (Mikwar et al., 2020). The present study demonstrated 48 meiosis-related genes differentially expressed in mature oocytes of advanced maternal age in comparison to young maternal age patients. Among them are *TUBB8,* which encodes the primary beta-tubulin subunit of oocytes and early embryos, and *BUB3*, which encodes a protein involved in spindle checkpoint function. *TUBB8* and *BUB3* were already associated with infertility due to oocyte meiotic arrest and mosaic variegated aneuploidy syndrome, respectively (GeneCards, 2022). Genes related to meiotic recombination, chromosome cohesion, spindle assembly checkpoint, post-translational modification of histones and tubulin, and mitochondrial functions, are great targets for research, since impairment in these mechanisms has been proposed to explain the higher incidence of oocyte aneuploidies in older females (Mikwar et al., 2020).

Aside from advanced maternal age, it was expected that alterations in meiosis-related gene expression could be associated with human reproductive disorders, considering their pattern of expression in male and female gonads in critical periods of gametogenesis. Nevertheless, no differences were observed in the oocytes’ gene expression of PCOS patients in comparison to the control group. PCOS is a multifactorial disorder, influenced by intrinsic and extrinsic factors, such as insulin resistance, hyperandrogenism, environmental factors, genetics, and epigenetics (Sadeghi et al., 2022). Therefore, as many factors are involved in the etiology of PCOS, the meiosis-related genes considered in the present study probably do not play a crucial role in the mechanisms behind PCOS etiology. Furthermore, a recent study on global gene expression of oocytes and cumulus cells from PCOS patients have not demonstrated the genes evaluated here as possible candidates (Li et al., 2021).

Contrastingly, when evaluating male reproductive disorders, 238 and 290 meiosis-related genes were differentially expressed in teratozoospermic and NOA patients in comparison to the control group, respectively. Most of these genes were downregulated in the conditions affecting human male reproduction, demonstrating that expression of meiosis-related genes may be necessaire in processes related to male fertility. Additionally, 145 genes were differentially expressed in both teratozoospermic and NOA patients compared to the control group, many of them already associated with spermatogenic failure and sperm maturation defect, according to our search for phenotype descriptions in the Ensembl database. Previous studies have demonstrated a downregulation profile in meiosis-related genes in both NOA and teratozoospermic patients (Fu et al., 2016; He et al., 2021). He et al. demonstrated 430 downregulated genes in NOA in comparison to the control group, many of them associated with spermatogenesis, multicellular organism development, cell differentiation, spermatid development, and sperm motility (He et al., 2021); Fu et al. demonstrated 2158 downregulated genes in teratozoospermic in comparison to normospermic patients, mostly related to reproduction, mitotic cell cycle, and spermatogenesis (Fu et al., 2016).

Considering the 17 meiosis-related genes differentially expressed in all the developmental periods analyzed, it was observed downregulation of *ADAD1, BRDT, GTSF1, HORMAD1, OOEP, SPATA22, SYCP3*, and *TEX11* in both teratozoospermic sperm and NOA testicle samples, evidencing a role in male reproduction. Except for *OOEP,* all the other downregulated genes have been linked to male infertility disorders, including NOA and teratozoospermia (Aarabi et al., 2006; Miyamoto et al., 2012; Chen et al., 2020; Greither et al., 2020; Snyder et al., 2020; Her et al., 2021; Ji et al., 2021; Wu et al., 2021; Omolaoye et al., 2022). OOEP is a member of the SCMC, a maternal complex essential to the progression beyond the first embryonic cell divisions (Li et al., 2008; Zhu et al., 2014). Although mutations in the SCMC genes are associated with female reproductive disorders (Demond et al., 2019; Zhang et al., 2019), a relationship between SCMC components and male reproduction was only recently suggested by (Feng et al., 2020), evidencing *Tle6* playing a role in the proliferation and cell cycle of the mouse spermatogonia (Feng et al., 2020).

Although no obvious association of *OOEP* with male reproduction the co-expression network performed for *OOEP* demonstrated the co-expression with genes located in the Y chromosome, related to human reproduction, spermatogenesis, and male infertility. Since co-expressed genes are simultaneously active (van Dam et al., 2018), downregulation of *OOEP* in patients with NOA and teratozoospermia could affect the entire gene expression regulation, even though the structure or even the protein expression might not be affected. Furthermore, *Ooep* has been related to processes involved in DNA repair (He et al., 2018; Zhao et al., 2018), and impairment in these mechanisms is associated with spermatogenesis arrest (Gunes et al., 2015), and teratozoospermia (Ammar et al., 2020).

This study has some limitations, such as not having the validation of gene expression results by qPCR and not demonstrating protein expression. However, all data used for analysis are from experimental studies of different datasets that pointed in the same direction. In this way, the results presented here contribute to the understanding of gene expression patterns involved in the differences between male and female meiosis, by (I) demonstrating that meiosis-related genes are differentially expressed in human male and female gonads throughout gametogenesis and (II) presenting altered gene expression in human male reproductive disorders and oocytes of advanced maternal age patients. Hence, concerning basic sciences and assisted reproduction application, future studies aiming to elucidate the molecular mechanisms of meiosis should consider the genes presented in this study. These results pinpoint to possible new candidate genes that could have clinical implications, therefore should be considered biomarkers in panels for patients with NOA or teratozoospermia.

## Data Availability

The original contributions presented in the study are included in the article/Supplementary Material, further inquiries can be directed to the corresponding author.
